# Inhibition of tumor necrosis factor signaling attenuates renal immune cell infiltration in experimental membranous nephropathy

**DOI:** 10.18632/oncotarget.22881

**Published:** 2017-12-04

**Authors:** Yen-Sung Huang, Shin-Huei Fu, Kuo-Cheng Lu, Jin-Shuen Chen, Hsin-Yi Hsieh, Huey-Kang Sytwu, Chia-Chao Wu

**Affiliations:** ^1^ Institute of Biomedical Sciences, Academia Sinica, Taipei, Taiwan; ^2^ Graduate Institute of Microbiology and Immunology, National Defense Medical Center, Taipei, Taiwan; ^3^ Division of Nephrology, Department of Medicine, Tri-Service General Hospital, National Defense Medical Center, Taipei, Taiwan; ^4^ Division of Nephrology, Department of Medicine, Fu Jen Catholic University Hospital, New Taipei City, Taiwan

**Keywords:** tumor necrosis factor, membranous nephropathy, etanercept, preligand assembly domain, immunomodulation

## Abstract

Idiopathic membranous nephropathy (MN) is an autoimmune-mediated glomerulonephritis and the most common cause of idiopathic nephrotic syndrome in adult humans. A tumor necrosis factor α (TNF-α)-mediated inflammatory response via TNF receptor 1 (TNFR1) and TNFR2 has been proposed as a pathogenic factor. In this study, we assessed the therapeutic response to blocking TNF signaling in experimental MN. Murine MN was induced experimentally by cationic bovine serum albumin (cBSA); phosphate-buffered saline was used in control mice. In MN mice, TNF was inhibited by etanercept blocking of TNFR1/TNFR2 or the preligand assembly domain fusion protein (PLAD.Fc), a small fusion protein that can preferentially block TNFR1 signaling. Disease severity and possible mechanisms were assessed by analyzing the metabolic and histopathology profiles, lymphocyte subsets, immunoglobulin production, oxidative stress, and apoptosis. cBSA-induced MN mice exhibited typical nephrotic syndrome and renal histopathology. MN mice given etanercept or PLAD.Fc did not exhibit significant reduction of proteinuria, amelioration of glomerular lesions, or attenuation of immune complex deposition. Immune cell subsets, serum immunoglobulin levels, production of reactive oxygen species, and cell apoptosis in the kidney were not altered by TNF inhibition. By contrast, MN mice receiving etanercept or PLAD.Fc exhibited significantly decreased infiltration of immune cells into the kidney. These results show that the therapeutic effects of blocking TNFR1 and/or TNFR2 signaling in experimental MN are not clinically effective. However, TNF signaling inhibition significantly attenuated renal immune cell infiltration in experimental MN.

## INTRODUCTION

Idiopathic membranous nephropathy (MN) is the most common cause of idiopathic nephrotic syndrome in adult humans and 30–40% of patients with MN will ultimately progress into end-stage renal failure after 10–15 years [1–3]. MN is an immune-mediated glomerulonephritis characterized by deposition of immune complexes within the subepithelial space, which initiates serial responses including complement activation, inflammation, oxidative injury, and apoptosis, that are central to the pathogenesis of MN [4–6]. During the process of human MN, there is an increased production of tumor necrosis factor ɑ (TNF-ɑ) in the serum. MN causes increased expression of inflammatory mediators, especially proinflammatory cytokines such as, interleukin 1β (IL-1β), IL-6 and TNF-ɑ, which appear to be important in the MN process [4–6]. Although present immunosuppressive treatment of MN has improved outcomes, the current therapies are not always effective and satisfied due to adverse effects [7, 8]. Therefore, there is still debate about the most appropriate therapeutics for MN.

TNF-ɑ is a pleiotropic cytokine produced mainly by immune cells such as macrophages, dendritic cells, and T lymphocytes, and is implicated in immune regulation [9–12]. TNF may bind to two distinct transmembrane receptors, TNF receptor 1 (TNFR1; p55 or CD120a) or TNFR2 (p75 or CD120b), which are differentially expressed on cells and tissues where they exert diverse biological effects including cell death, survival, differentiation, proliferation, migration, and inflammation. TNFR1 appears to play a predominantly proinflammatory role, whereas TNFR2 may play an important role in disease immunoregulation [9–12]. Certain TNF-ɑ gene polymorphisms are associated with an elevated risk for the development of MN [13, 14]. The serum and urinary TNF-ɑ levels increase in MN patients, and urinary TNF-ɑ excretion correlates positively with proteinuria in MN patients [15, 16]. Taken together, these findings support the idea that TNF-ɑ may play an important role as a pathogenic mediator in MN. We reasoned that TNF-ɑ inhibition may be a potential therapeutic target for MN treatment.

Previous results of blocking TNF as an MN treatment are conflicting [17, 18]. Simultaneously or specifically blocking TNFR1 and TNFR2 may reveal the divergent functions of TNFR1 and TNFR2, which may be useful for developing a specific TNFR-directed therapeutic strategy [12, 19–22]. The preligand assembly domain (PLAD) is the overlapping region of cysteine-rich domains of the TNFR, which is necessary and sufficient for mediating the receptor response [23–25]. PLAD.Fc is a small fusion protein that may preferentially and specifically block TNFR1 signal transduction [23–25]. In this study, we treated MN mice by inhibiting TNF using etanercept blocking of TNFR1/TNFR2 or PLAD.Fc, a small fusion protein that can preferentially block TNFR1 signaling.

## RESULTS

### Effects of inhibition of TNF on proteinuria and renal histology

**Figure 1:**
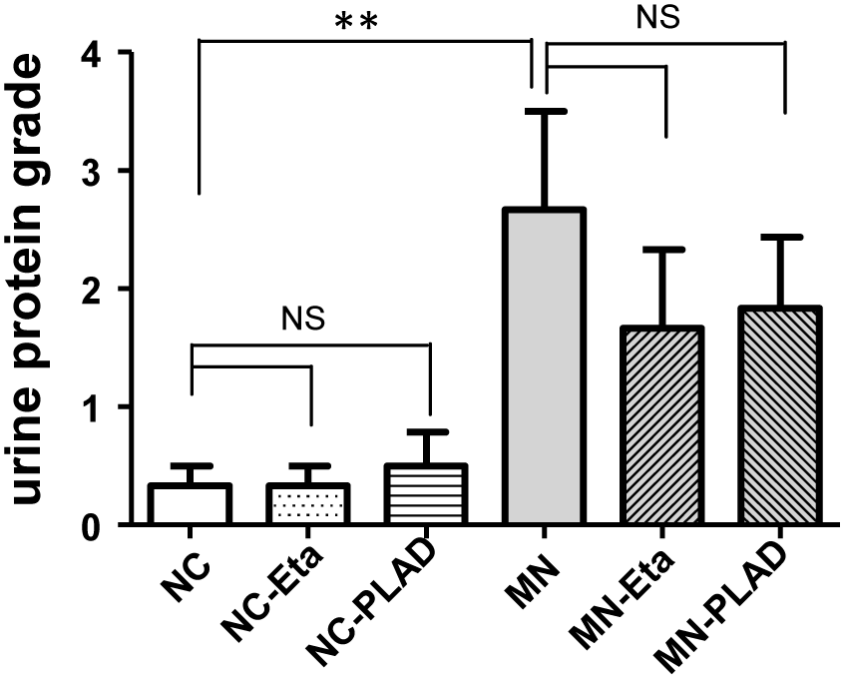
Effects of TNF blockade on proteinuria in mice with experimental MN Urinary proteinuria of the normal control group (NC), membranous nephropathy group (MN), mice with membranous nephropathy receiving etanercept (MN-Eta) and mice with membranous nephropathy receiving preligand assembly domain fusion protein (MN-PLAD) were analyzed serially with urine strips. ^*^p < 0.05 versus the control group. ^**^p < 0.05 versus the MN group.

The cBSA-induced MN mice exhibited typical heavy proteinuria. MN mice from the groups given etanercept or PLAD.Fc did not exhibit significant reduction in proteinuria. No proteinuria was noted in NC mice receiving etanercept or PLAD.Fc (Figure 1).

**Figure 2:**
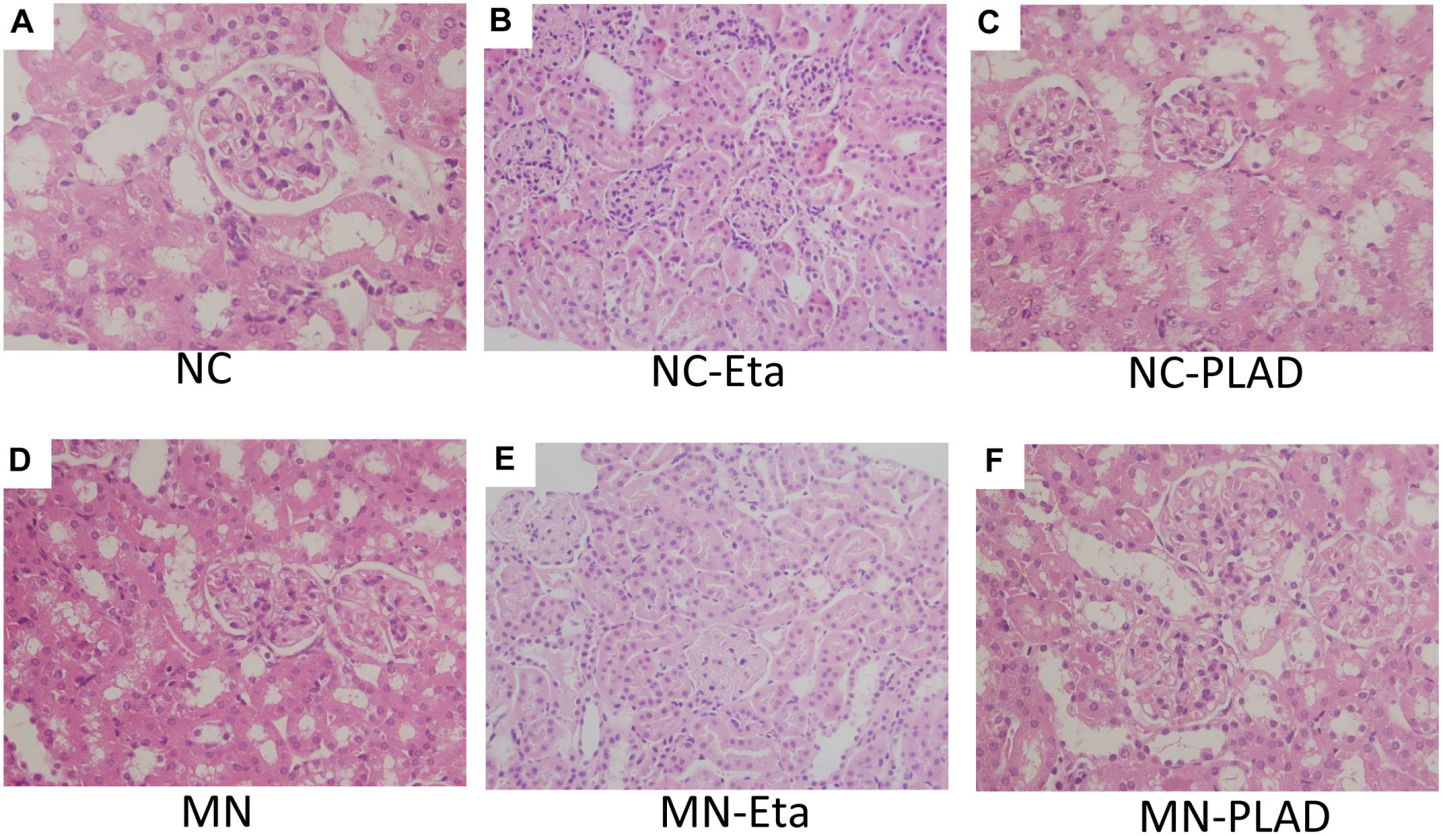
Changes in renal histopathology in mice with experimental MN as shown by H&E staining Mice in the NC group **(A–C)** and MN group **(D–F)** were treated with PBS (A and D), etanercept (MN-Eta; B and E), or preligand assembly domain fusion protein (MN-PLAD; C and F) and the tissues were stained with H&E. All images are at 400× magnification.

H&E staining showed typical renal histopathology of diffuse thickening of the glomerular basement membrane in the cBSA-induced MN mice. MN-etanercept or PLAD.Fc mice exhibited a similar severity in pathology (Figure 2D–2F); by contrast, no significant changes were observed in NC mice that received etanercept or PLAD.Fc (Figure 2A–2C).

**Figure 3:**
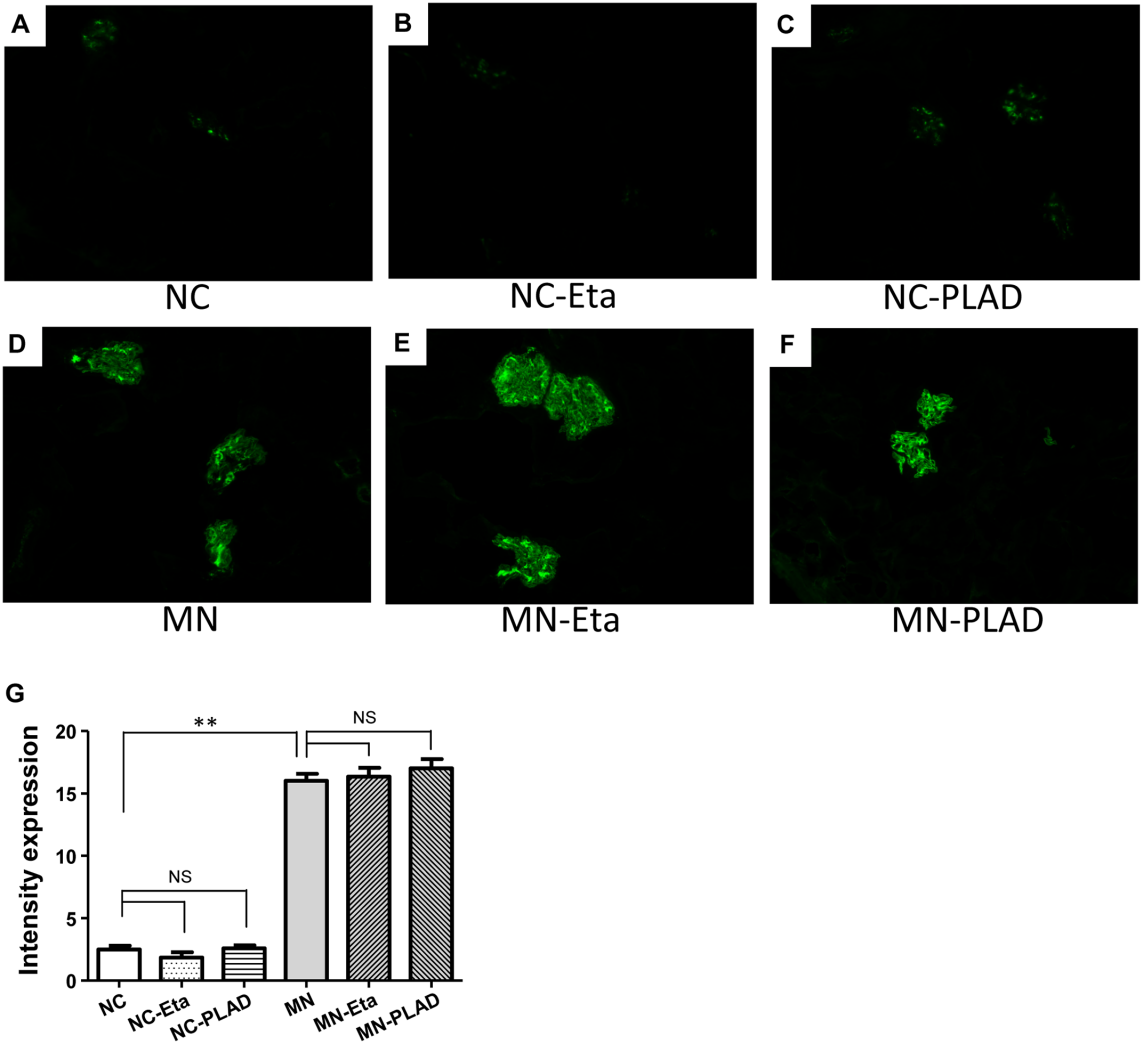
Changes in renal histopathology in mice with experimental MN shown by immunofluorescence staining for IgG Mice in the NC group **(A–C)** and MN group **(D–F)** were treated with PBS (A and D), etanercept (MN-Eta; B and E), or preligand assembly domain fusion protein (MN-PLAD; C and F), and quantitative data for immunofluorescence staining of IgG are shown in **(G)**.

**Figure 4:**
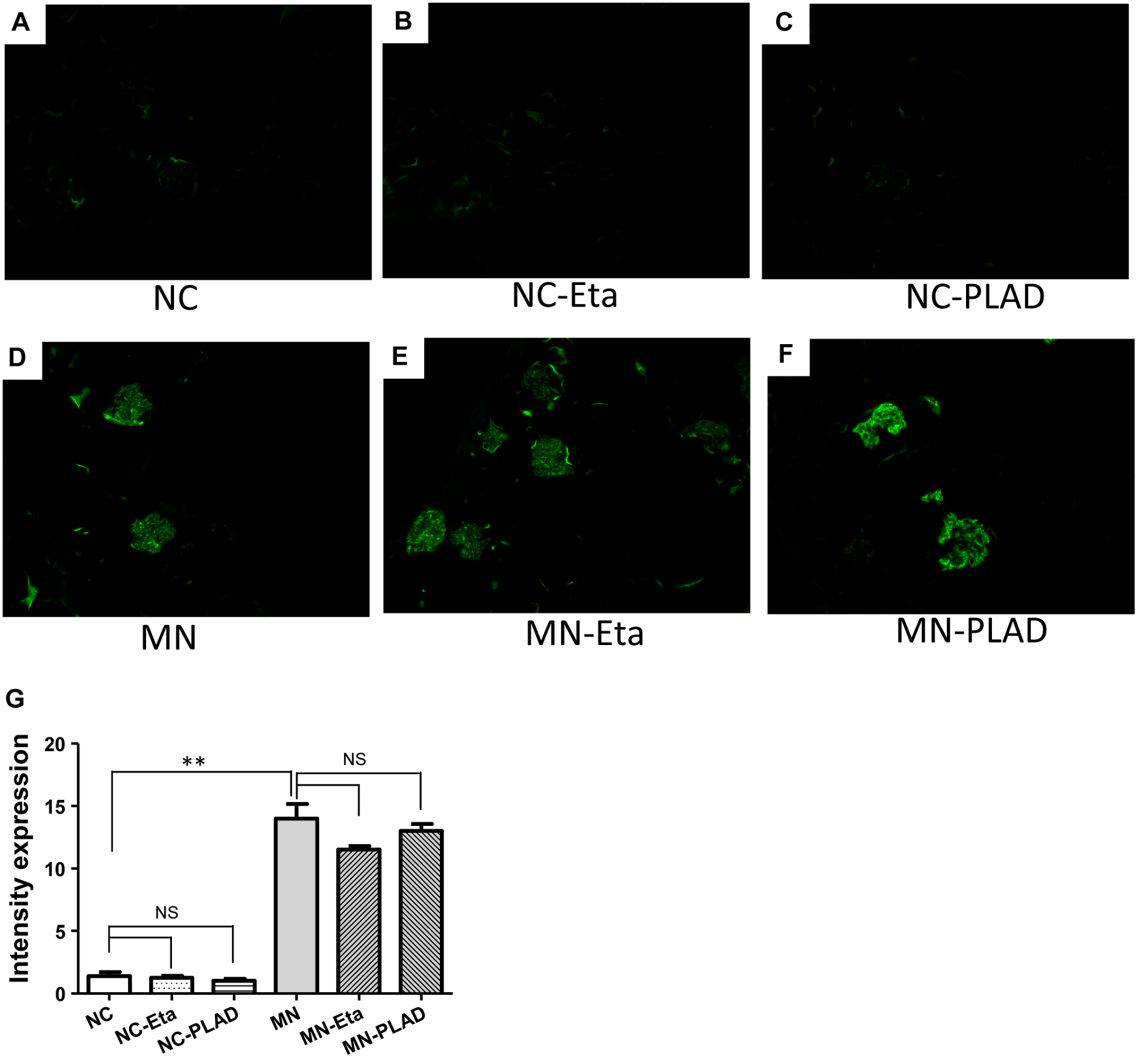
Changes in renal histopathology of mice with experimental MN as shown by immunofluorescence staining for C3 The NC group **(A–C)** and MN group **(D–F)** were treated with PBS (A and D), etanercept (MN-Eta; B and E), or preligand assembly domain fusion protein (MN-PLAD; C and F). Quantitative data for immunofluorescence staining of C3 are shown in **(G)**.

MN mice exhibited significant granular immunofluorescence staining for IgG, which showed as a discrete beaded appearance along the glomerular capillary wall. This pattern was similar in the MN- etanercept and MN-PLAD mice, which suggested that inhibition of TNF did not change the deposition of immune complexes (Figure 3A–3G). Immunofluorescence staining for C3 also showed a similar pattern as for IgG fluorescence in MN mice; inhibition of TNF did not attenuate this response (Figure 4A–4G). The histopathological and immunofluorescence features did not differ between the NC- etanercept, NC-PLAD, and NC mice.

### Effect of inhibition of TNF on lymphocyte subsets

**Figure 5:**
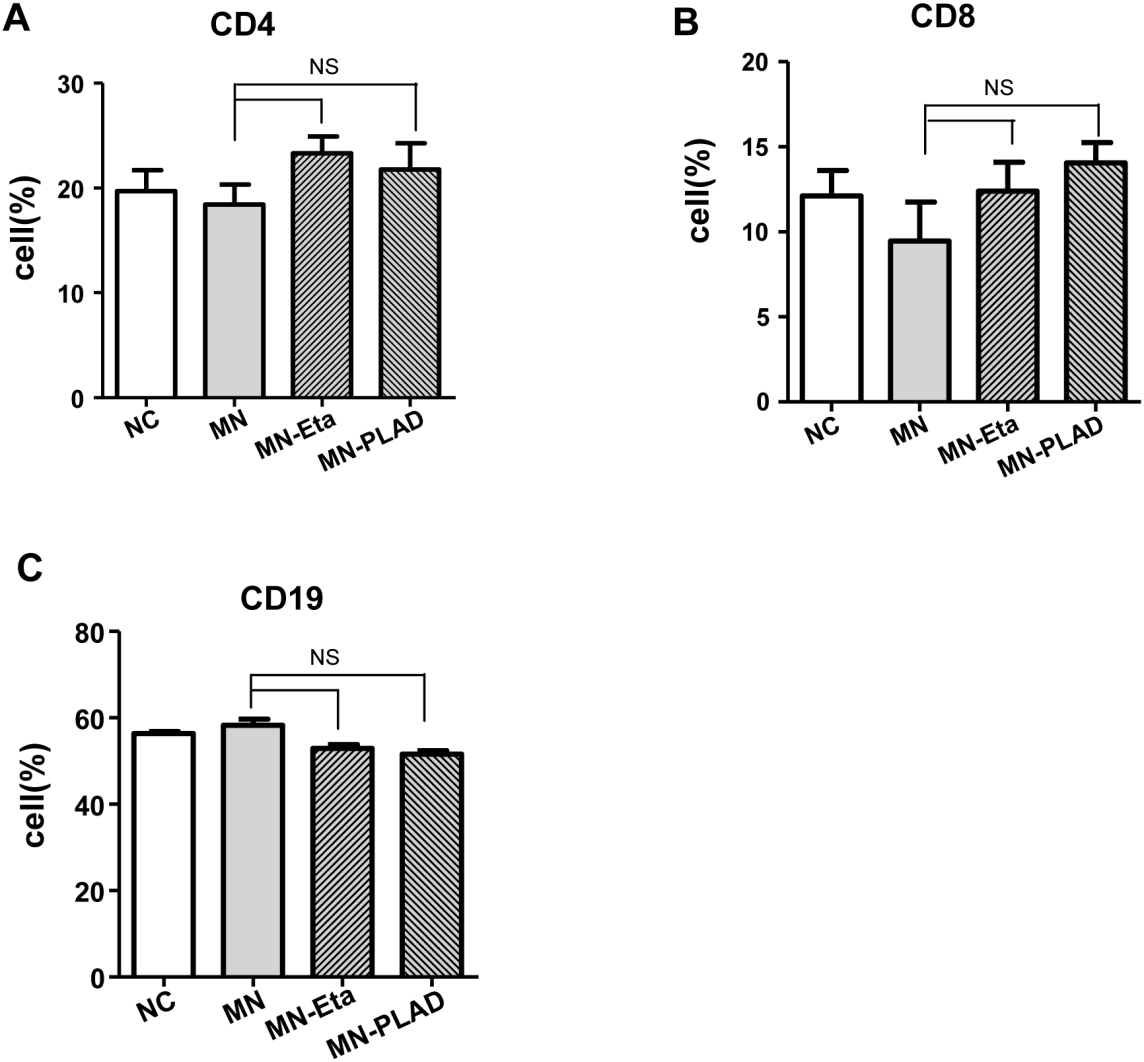
Distribution of lymphocyte subsets in spleens from mice with experimental MN The percentages of immune cells including **(A)** CD4, **(B)** CD8, and **(C)** CD19 as assessed by flow cytometry did not change significantly in mice from the NC, MN, and MN groups treated with etanercept (Eta) or preligand assembly domain fusion protein (PLAD).

Immune cells play important roles in pathogenesis of MN. We examined used flow cytometry to examine the effects of TNF inhibition on splenic lymphocytes. The lymphocyte subsets of CD4^+^ T cells, CD8^+^ T cells, and CD19^+^ B cells did not differ significantly between mice in the NC, MN, MN-etanercept, and MN-PLAD groups (Figure 5A–5C).

### Effects of inhibition of TNF on ROS production and apoptosis

**Figure 6:**
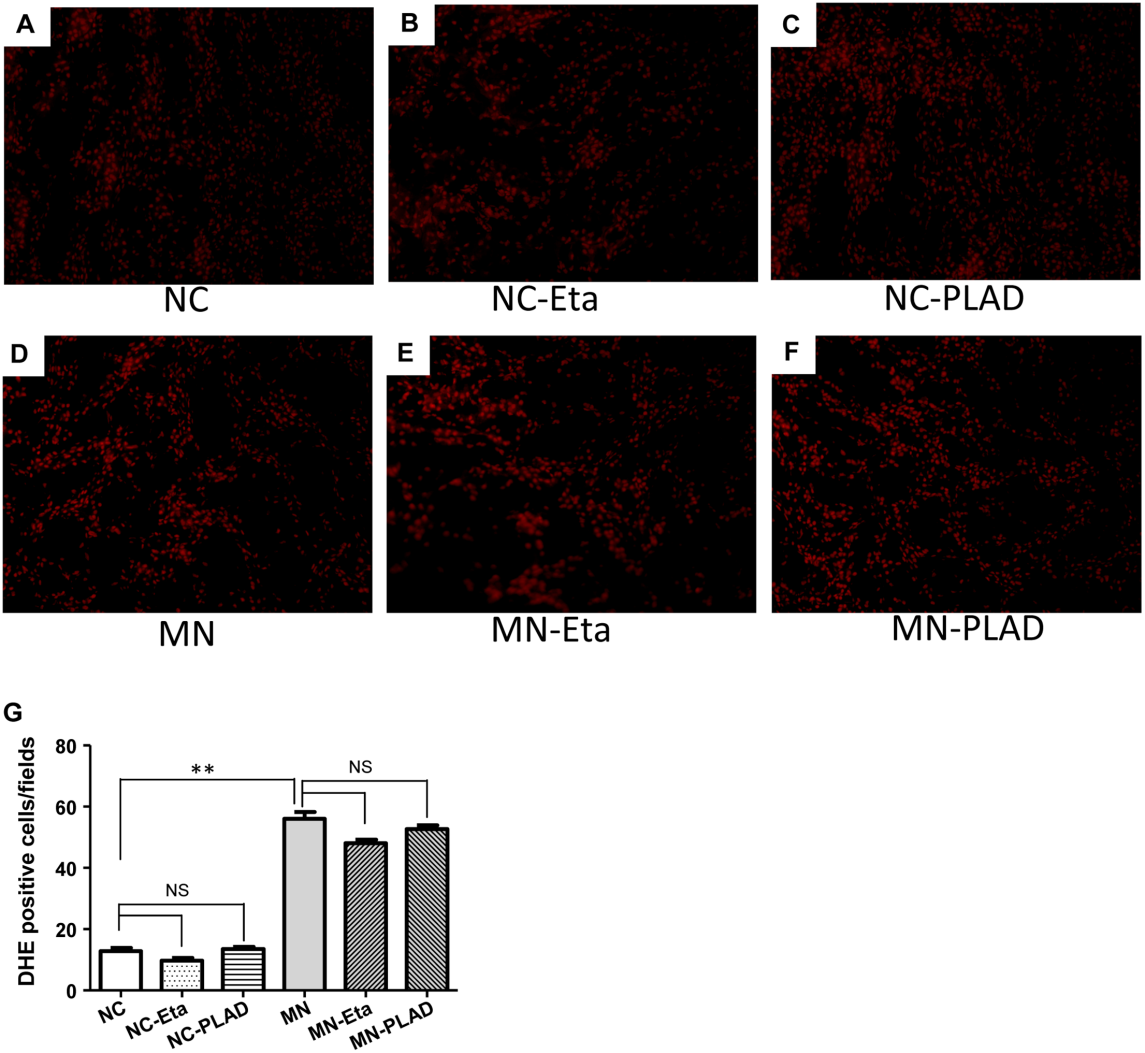
Superoxide anion production in kidney cells Fluorescence micrographs of DHE-positive cells in the kidneys of mice from the NC group **(A–C)** and MN group **(D–F)**, which were treated with PBS (A and D), etanercept (MN-Eta; B and E), or preligand assembly domain fusion protein (MN-PLAD; C and F) as shown by immunofluorescence staining for DHE. Quantitative data are shown in **(G)**. All images are at 400× magnification. ^*^p < 0.05 versus the control group. ^**^p < 0.05 versus the MN group.

Oxidative stress has been shown to play an important role in the development and progression of MN. We detected the production of superoxide anion radical in kidneys (Figure 6A–6G). The level of DHE fluorescence was low in NC mice and was significantly higher in MN mice; no attenuation of fluorescence was observed in MN mice treated with etanercept or MN-PLAD. These findings suggested that there are increased production of ROS in MN kidneys and that inhibition of TNF did not attenuate this effect.

TNF is associated with apoptotic effects, and we also checked its effect on apoptosis in renal tissues of MN mice (data not shown). MN mice showed an increased number of apoptotic cells in the kidneys compared with NC mice, and inhibition of TNF treatment did not attenuate this effect. This finding suggested that the antiapoptotic effect of TNF inhibition does not have a therapeutic effect against MN.

### Effects of inhibition of TNF on immune cell infiltration in kidney

**Figure 7:**
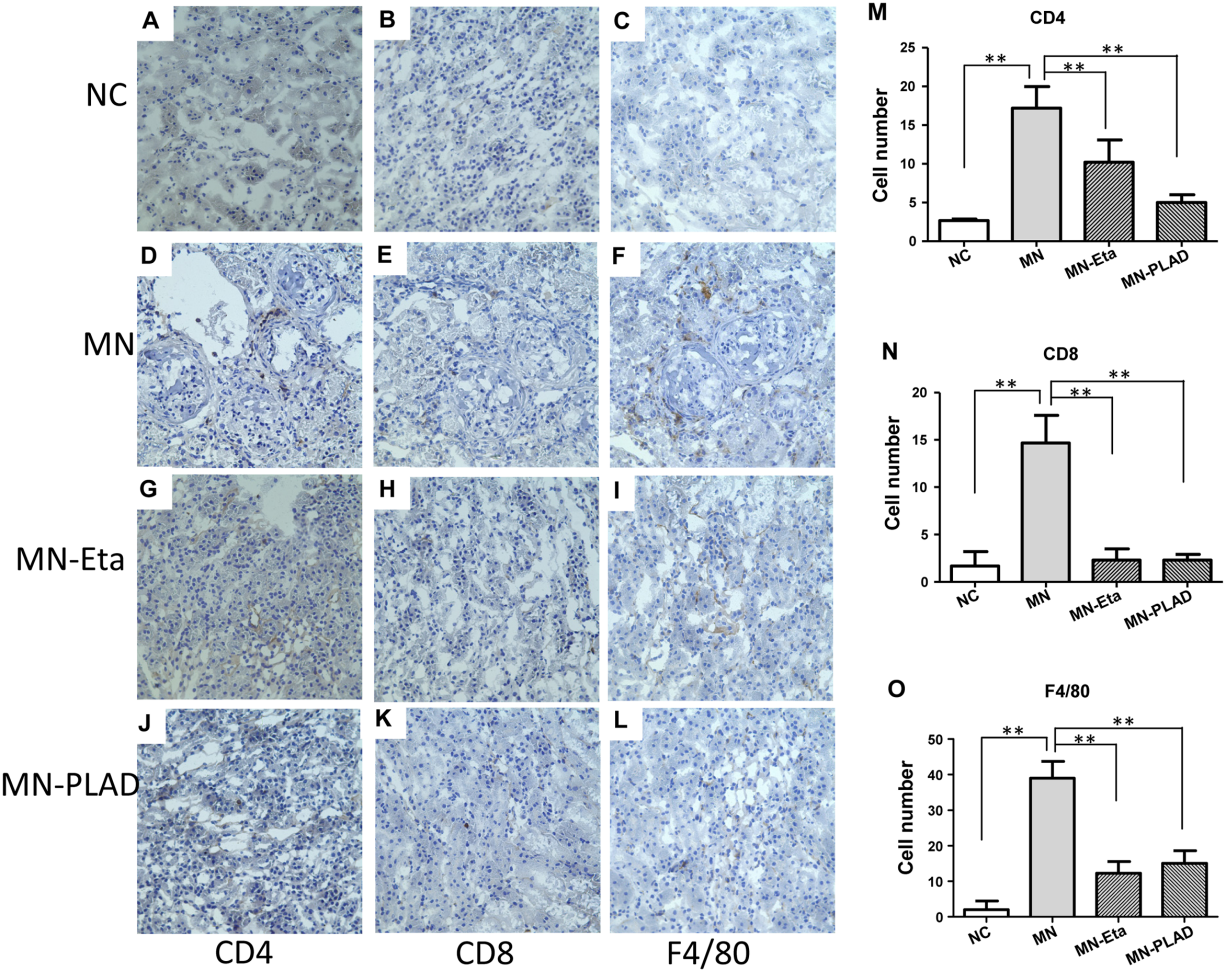
Immunohistochemistry for CD4, CD8, CD19, and F4/80 in kidneys Kidneys from mice in the NC group **(A–C)** and MN group **(D–L)**, which were treated with PBS (D-F), etanercept (MN-Eta; G-I), or preligand assembly domain fusion protein (MN-PLAD; J-L) were stained for anti-CD4 (A, D, G, J), anti-CD8 (B, E, H, K), and anti-F4/80 (C, F, I, L). Quantitative data are shown in **(M-O**). All images are at 400× magnification. ^*^p < 0.05 versus the control group.

To investigate the effects of inhibition of TNF on immune cell infiltration in kidney in cBSA-induced MN, we used IHC to identify renal immune cells in mice from the NC group (A–C), and MN group (D–L) receiving PBS (D-F), etanercept (MN-Eat; G-I) and PLAD.Fc (MN-PLAD; J-L). Subgroups of immune cells including CD4^+^ T cells (Figure 7A, 7D, 7G, 7J), CD8^+^ T cells (Figure 7B, 7E, 7H, 7K), and F4/80 monocytes (Figure 7C, 7F, 7I, 7L) were analyzed. F4/80^+^ macrophages were the most abundant inflammatory cells found in glomeruli. By contrast, both the MN-Eta and MN-PLAD groups showed significantly less infiltration of immune cells.

## DISCUSSION

MN mice in groups receiving etanercept or PLAD.Fc did not exhibit significant reduction of proteinuria, amelioration of glomerular lesions, or attenuation of immune complex deposition. There were also no effects on the activation of complement, immune cell subsets, serum Ig level, superoxide level, or degree of apoptosis in kidney tissue. However, significantly less infiltration of immune cells was noted in MN mice receiving etanercept or PLAD.Fc. These results show that the therapeutic effects of blocking TNFR1 and/or TNFR2 signaling in experimental MN are not effective. The role of attenuated immune cell infiltration in the kidney has not been determined.

In our study, TNF blocking did not improve the clinical outcomes of MN, and the only effect was a decrease in immune cell infiltration in the kidney as shown histologically. The deposited immune complexes subsequently induce complement activation, and oxidative injury, are involved during MN disease progression [4–6]. Because immune complex deposition and activation of complement and ROS occur to the same extent, these may in part explain the similar glomerular damage and clinical picture in MN. TNF-ɑ exerts diverse biological effects and is implicated in the inflammatory cascade leading to renal injury [12, 19, 21]. High TNF-ɑ level has been reported in patients with MN, and the TNFA2 and TNFd2 alleles are strongly associated with the occurrence or initiation of MN [13, 15]. These findings support the idea that TNF-ɑ plays a pathogenic role in MN.

Experimental models of glomerulonephritis have shown that TNF-ɑ has contrasting proinflammatory and immunosuppressive roles in the kidney. It appears that TNFR1 plays a predominantly proinflammatory role, whereas TNFR2 plays an important role in disease immunoregulation [12, 22]. Inhibition of TNF-ɑ through the TNFR1 pathway by itself or combined with inhibition of TNFR2 signaling markedly decreases the recruitment of glomerular and tubulointerstitial infiltration of immune cells including T cells, B cells, and F4/80 macrophages [12, 22]. Proteinuria itself can induce immune cell infiltration in MN. The same degree of proteinuria observed in our study suggests that this phenomenon is proteinuria independent, but possibly TNF-ɑ associated. These infiltrates may be independent of glomerular immune complex deposition and/or the resulting peritubular hypoxia. Presumably periglomerular macrophages attracted by locally produced chemokines or activated by inflammatory mediators [12, 21, 22]. By contrast, TNF inhibition does not appear to alter the systemic response that regulates lymphocyte activation or to prevent the accumulation of renal lymphoid infiltrates. Our results suggest that TNF is a critical cytokine in the renal effector response to glomerular immune complex deposition.

TNF-ɑ is synthesized predominantly by infiltrating T cells and monocytes/macrophages. This cytokine can amplify the renal inflammatory response via increasing the expression of adhesion molecules and inducing the cells to release growth factors, other cytokines, and proinflammatory chemokines in an autocrine and paracrine manner [12, 21, 22]. Because both the infiltrating and intrinsic glomerular cells can synthesize TNF-ɑ, these cells are considered equally important for glomerular injury. Morevoer, TNF-ɑ can directly activate NADPH oxidase, which leads to the local generation of ROS via phosphodiesterase-dependent mechanisms.

Although TNF-ɑ has both immunosuppressive and proinflammatory properties, it is not simple to predict the effect of anti-TNF-ɑ treatment in MN [12, 21, 22]. In our study, etanercept treatment did not significantly alter the clinical and histological severity, although it reduced immune cell infiltration. Our results are consistent with those of a previous study [17] in which etanercept was used to inhibit TNF-ɑ in MN patients with nephrotic syndrome for 3 months but did not produce any significant clinical benefit. Previous studies suggest that specifically targeted therapeutics that focus on inhibiting individual TNFR1 or TNFR2, may help balance the proinflammatory and immunomodulatory functions of TNF in renal disease and thus represent potential therapeutics with a better clinical outcome [12, 21]. Therefore, we used purified recombinant PLAD.Fc, which is specific to the TNFR1, to effectively block TNFR1 signaling. There were no significant differences in outcomes of blocking TNFR1 or TNFR2 by etanercept and specifically blocking the TNFR1 by PLAD60.

Several factors differentiate MN from other clinical entities and might be involved in the absence of effects in the present trial. It is possible that a longer course of etanercept from an earlier stage of MN would have been clinically effective. The treatment used in our study was initiated very early at the start of MN and lasted the whole process of the disease, but was not effective in preventing the clinical signs of MN. These data do not support the role of TNF in MN and may indicate that the TNF may be a responder rather than a contributor to the MN process. By contrast, pentoxifylline treatment in a similar model significantly decreased proteinuria and caused a high rate of remission, as shown by decreased plasma and urinary TNF-ɑ levels [18]. Although pentoxifylline suppresses the endogenous production of TNF-ɑ by monocytes/macrophages, additional circumstantial data support a role of this drug in glomerular disease. The antiproteinuric effects of pentoxifylline might be attributed to its rheological actions, which increase the deformability of erythrocytes and reduce blood viscosity, and glomerular hydraulic pressure. On the other hand, pentoxifylline is an antagonist of adenosine, and may reduce hyperfiltration and proteinuria through blockade of adenosine receptors. Hence, the therapeutic effect of pentoxifylline for MN may be beyond that of TNF-ɑ.

A diversity of effects has been reported across various autoimmune diseases, which involve different pathogenic mechanisms and the involvement of organs and/or systems. Anti-TNF-ɑ treatments demonstrate great benefit in autoimmune diseases such as rheumatoid arthritis and Crohn’s disease. There is currently no validated indication for anti-TNF-ɑ treatments in kidney diseases. Some studies have suggested benefits of anti-TNF-ɑ treatments to improve the extent of renal damage in antineutrophil cytoplasmic antibody-associated systemic vasculitis [26]. Some data suggest a potential benefit of infliximab to improve lupus nephropathy. TNF-ɑ antagonist treatment reduces albuminuria and prevents completely the development of crescents in the heterologous rat model of anti-GBM glomerulonephritis. Anti-TNF-ɑ treatments or TNF-deficient mice are partially protected against glomerular injury through lower renal infiltration of T cells and neutrophils in experimental models of glomerulonephritis [22, 26]. TNF-ɑ is believed to play a central role during the progression of diabetic nephropathy [21]. Experimental studies have shown the beneficial actions on the kidney attributed to TNF-ɑ inhibition; however, these are at present limited to pentoxifylline administration in patients with chronic kidney disease because of its significant beneficial effects.

A previous study showed that 3 months of therapy with etanercept was safe and well tolerated by MN patients [18]. Anti-TNF-ɑ treatments are generally safe in comparison with traditional immunosuppressive drugs. However, the potential side effects of anti-TNF-ɑ treatment should be considered, such as the elevated incidence of bacterial infections, activation of latent tuberculosis infection, and the increased incidence of lymphoma and solid tumors during anti-TNF-ɑ treatment. Anti-TNF treatment can also occasionally increase the risk for induction of autoimmune symptoms and is associated with the induction of antibodies to double-stranded DNA. Furthermore, some rheumatoid arthritis patients undergoing therapy with anti-TNF-ɑ agents develop new-onset glomerular disease caused by pauci-immune necrotizing and crescentic glomerulonephritis, membranous nephropathy, or IgA nephropathy [27–29]. The inefficiency and potentially harmful effects of anti-TNF-ɑ agents raise concerns about the use of this treatment in MN patients.

PLAD occurs in the region overlapping the first cysteine-rich domain of P60 and P80 TNFRs and Fas, which are sufficient for mediating specific ligand-independent receptor assembly and subsequent signaling [23–25]. Targeting PLAD with a soluble form of PLAD recombinant protein (PLAD.Fc) preferentially blocks the TNFR1 signal by interfering with its trimerization, and this may offer another approach for specifically treating TNFR1 signal-causing diseases [23–25]. Using the recombinant PLAD.Fc protein to block TNFR1 assembly, we previously showed that PLAD.Fc treatment significantly reduces the production of TNFR1-driving proinflammatory cytokines and protects from autoimmune diabetes in an experimental model using nonobese diabetic mice [30]. We also targeted PLAD of TNFR1 to interfere with receptor trimerization blocking downstream signaling and found that this protected against Th17-mediated colitis by boosting the Th2 response in BLIMP-1-knockout mice [31]. We used this strategy instead of using TNFR1/2-knockout mice because the induction of experimental cBSA-induced MN is not possible in mice with a B6 background.

Urinary TNF-ɑ excretion correlates with proteinuria in patients with MN and TNF-ɑ gene polymorphism is a risk factor for development of MN. One study demonstrated that the circulating level of the TNFR at the time of initial diagnosis may provide a biomarker for the prediction of renal progression in patients with MN [16]. Furthermore, elevated concentrations of circulating TNF-ɑ, TNFR1, and TNFR2 are associated with loss of renal function and may be predictors of the progression of diabetic nephropathy to stage 3 chronic kidney disease or end-stage renal disease. Hence, TNF may be more suitable as a biomarker than as a therapy in the treatment of MN.

The develop an ideal therapeutic agents effectively and specifically for MN treatment is an important issue to nephrologists. The therapeutic effects of anti-TNF have been demonstrated in many human inflammatory and autoimmune diseases such as rheumatoid arthritis; however, our results did not show therapeutic effects in MN. We assume that anti-TNF does not affect the key pathogenic responses such as the production of Igs and subsequent immune complex deposition, inflammation, complement activation, oxidative stress, and apoptosis. We conclude that anti-TNF may not be a suitable MN treatment.

## MATERIALS AND METHODS

### Mice

All experiments were conducted according to the National Institutes of Health Guidelines, and all animal experiments were approved by the Animal Care and Use Committee of the National Defense Medical Center, Taipei, Taiwan (IACUC-11-149).

### Experimental design

The experimental MN model was induced as previously described [32–37]. The mice were randomly assigned to the experimental (MN) group or the control (NC) group. After immunized with cationic bovine serum albumin (cBSA) emulsified in complete Freund’s adjuvant, the MN group further received cBSA (13 mg/kg) intravenously trice per week for 6 weeks, and the NC group received phosphate-buffered saline (PBS) instead as the same schedule. Both groups mice were then randomly assigned to three subgroups (*n* = 5 in each subgroup), each of which received one of three treatments: intravenous injection of TNFR preligand assembly domain fusion protein (PLAD.Fc, 5 mg/kg) twice a week; subcutaneous injection of etanercept (Eta, 0.8 mg/kg) once a week (MN-PLAD, MN-Eta, NC-PLAD, and NC-Eta, respectively); or saline (MN and NC, respectively) since the time of MN induction. Homogeneous cBSA, construction of the plasmid and purification of the recombinant PLAD.Fc fusion protein were prepared as previously described [32, 33, 36–39]. Disease severity was verified by clinical metabolic profiles and by histopathologies, as described below.

### Serum and urine measurements

Urine samples were obtained for proteinuria screening using Labstix (Bayer Corp., Pittsburgh, PA, USA). Samples were group into five grades based on urine protein concentrations: 0 = 0-30 mg/dL, 1+, 30–100 mg/dL; 2+, 100–300 mg/dL; 3+, 300–1000 mg/dL; 4+, ≥ 2000 mg/dL.

### Histological studies of renal tissues and immunohistochemistry

Renal tissue samples were prepared by snap-freezing, then fixed with acetone for immunofluorescence (IF) or 10% formalin for hematoxylin and eosin (H&E) and immunohistochemistry (IHC). HE staining, IF and IHC were performed as previously described [32–37]. Fluorescein isothiocyanate-conjugated goat anti-mouse IgG and C3 (Capple, Durham, NC, USA) were used to incubate with frozen section. For immunohistochemistry, frozen sections were incubated with primary antibodies (anti-mouse CD4, CD8, and F4/80; BD Biosciences), stained with the Vectastain Elite ABC kit (Vector Lab, Burlingame, CA, USA), and developed using DAB (brown precipitate, Vector Lab). Slides were observed using a fluorescence and light microscope (Olympus, Tokyo, Japan).

### Flow cytometry

Splenocytes were isolated and stained with marker-specific antibodies: allophycocyanin (APC)-conjugated anti-mouse CD4, phycoerythrin (PE)-conjugated anti-mouse CD8, and FITC-conjugated anti-mouse CD19. All monoclonal antibodies were purchased from BD Biosciences (San Jose, CA, USA) or eBioscience (San Diego, CA, USA). The stained cells were analyzed by a FACSCalibur cell sorter and CellQuest software (Becton Dickinson, Franklin Lakes, NJ, USA).

### Reactive oxygen species (ROS) detection in the kidney

ROS production was determined with dihydroethidium (DHE; Molecular Probes, Eugene, OR, USA) labeling, as described previously [32–37]. Briefly, the frozen sections were incubated with DHE in a humidified chamber and the fluorescent images were quantified by counting the percentage of positive cells per kidney cross section.

### Terminal deoxynucleotidyl transferase-mediated nick end-labeling (TUNEL) assay

Apoptosis was assessed using TUNEL assay and *In Situ* Cell Death Detection Kit (Roche Molecular Biochemicals, Mannheim, Germany) as previously described [32–37]. Briefly, renal sections were fixed with 4% paraformaldehyde and washed with PBS. Then, cells were permeabilized with 0.1% Triton X-100, rinsed with PBS, incubated in the TUNEL reaction mixture and finally were observed with a fluorescence photomicroscope (Olympus, Tokyo, Japan).

### Statistical analysis

Data are expressed as mean ± standard deviation. The data were analyzed using one-way analysis of variance for multiple comparisons, and the Bonferroni test was used to correct for between-group differences. Significance (p ≤ 0.05) was ascertained using SPSS/PC (SPSS Inc., Chicago, IL, USA).

## Abbreviations

MN: membranous nephropathy
TNF-α: tumor necrosis factor α
TNFR1: tumor necrosis factor receptor 1
TNFR2: tumor necrosis factor receptor 2
cBSA: cationic bovine serum albumin
PLAD.Fc: preligand assembly domain fusion protein
Eta: etanercept
NC: normal control
PBS: phosphate-buffered saline
IF: immunofluorescence
H&E: hematoxylin and eosin
IHC: immunohistochemistry
APC: allophycocyanin
PE: phycoerythrin
ROS: Reactive oxygen species
DHE: dihydroethidium
TUNEL: Terminal deoxynucleotidyl transferase-mediated nick end-labeling

## References

[1] Glassock RJ (2003). Diagnosis and natural course of membranous nephropathy. Semin Nephrol.

[2] Cattran DC (2001). Idiopathic membranous glomerulonephritis. Kidney Int.

[3] Couser WG (2017). Primary Membranous Nephropathy. Clin J Am Soc Nephrol.

[4] Couser WG, Abrass CK (1988). Pathogenesis of membranous nephropathy. Annu Rev Med.

[5] Nangaku M, Shankland SJ, Couser WG (2005). Cellular response to injury in membranous nephropathy. J Am Soc Nephrol.

[6] Ronco P, Debiec H (2006). New insights into the pathogenesis of membranous glomerulonephritis. Curr Opin Nephrol Hypertens.

[7] du Buf-Vereijken PW, Branten AJ, Wetzels JF (2005). Idiopathic membranous nephropathy: outline and rationale of a treatment strategy. Am J Kidney Dis.

[8] Glassock RJ (2004). The treatment of idiopathic membranous nephropathy: a dilemma or a conundrum?. Am J Kidney Dis.

[9] Vassalli P (1992). The Pathophysiology of Tumor Necrosis Factors. Annu Rev Immunol.

[10] Ruiz-Ortega M, Ortiz A, Ramos AM (2014). Tumor necrosis factor-like weak inducer of apoptosis (TWEAK) and kidney disease. Curr Opin Nephrol Hypertens.

[11] Ernandez T, Mayadas TN (2009). Immunoregulatory role of TNFalpha in inflammatory kidney diseases. Kidney Int.

[12] Al-Lamki RS, Mayadas TN (2015). TNF receptors: signaling pathways and contribution to renal dysfunction. Kidney Int.

[13] Thibaudin D, Thibaudin L, Berthoux P, Mariat C, Filippis JP, Laurent B, Alamartine E, Berthoux F (2007). TNFA2 and d2 alleles of the tumor necrosis factor alpha gene polymorphism are associated with onset/occurrence of idiopathic membranous nephropathy. Kidney Int.

[14] Bantis C, Heering PJ, Aker S, Siekierka M, Kuhr N, Grabensee B, Ivens K (2006). Tumor necrosis factor-alpha gene G-308A polymorphism is a risk factor for the development of membranous glomerulonephritis. Am J Nephrol.

[15] Idasiak-Piechocka I, Oko A, Pawliczak E, Kaczmarek E, Czekalski S (2010). Urinary excretion of soluble tumour necrosis factor receptor 1 as a marker of increased risk of progressive kidney function deterioration in patients with primary chronic glomerulonephritis. Nephrol Dial Transplant.

[16] Lee SM, Yang S, Cha RH, Kim M, An JN, Paik JH, Kim DK, Kang SW, Lim CS, Kim YS, Lee JP (2014). Circulating TNF receptors are significant prognostic biomarkers for idiopathic membranous nephropathy. PloS One.

[17] Lionaki S, Siamopoulos K, Theodorou I, Papadimitraki E, Bertsias G, Boumpas D, Boletis J (2009). Inhibition of tumour necrosis factor alpha in idiopathic membranous nephropathy: a pilot study. Nephrol Dial Transplant.

[18] Ducloux D, Bresson-Vautrin C, Chalopin J (2001). Use of pentoxifylline in membranous nephropathy. Lancet.

[19] Al-Lamki RS, Wang J, Vandenabeele P, Bradley JA, Thiru S, Luo D, Min W, Pober JS, Bradley JR (2005). TNFR1- and TNFR2-mediated signaling pathways in human kidney are cell type-specific and differentially contribute to renal injury. FASEB J.

[20] Pavkov ME, Nelson RG, Knowler WC, Cheng Y, Krolewski AS, Niewczas MA (2015). Elevation of circulating TNF receptors 1 and 2 increases the risk of end-stage renal disease in American Indians with type 2 diabetes. Kidney Int.

[21] Pavkov ME, Weil EJ, Fufaa GD, Nelson RG, Lemley KV, Knowler WC, Niewczas MA, Krolewski AS (2016). Tumor necrosis factor receptors 1 and 2 are associated with early glomerular lesions in type 2 diabetes. Kidney Int.

[22] Vielhauer V, Stavrakis G, Mayadas TN (2005). Renal cell-expressed TNF receptor 2, not receptor 1, is essential for the development of glomerulonephritis. J Clin Invest.

[23] Deng GM, Zheng L, Chan FKM, Lenardo M (2005). Amelioration of inflammatory arthritis by targeting the pre-ligand assembly domain of tumor necrosis factor receptors. Nat Med.

[24] Chan FK, Chun HJ, Zheng L, Siegel RM, Bui KL, Lenardo MJ (2000). A Domain in TNF Receptors That Mediates Ligand-Independent Receptor Assembly and Signaling. Science.

[25] Jiang Y, Woronicz JD, Liu W, Goeddel DV (1999). Prevention of Constitutive TNF Receptor 1 Signaling by Silencer of Death Domains. Science.

[26] Little MA, Bhangal G, Smyth CL, Nakada MT, Cook HT, Nourshargh S, Pusey CD (2006). Therapeutic effect of anti-TNF-α antibodies in an experimental model of anti-neutrophil cytoplasm antibody-associated systemic vasculitis. J Am Soc Nephrol.

[27] Stokes MB, Foster K, Markowitz GS, Ebrahimi F, Hines W, Kaufman D, Moore B, Wolde D, D'Agati VD (2005). Development of glomerulonephritis during anti-TNF-alpha therapy for rheumatoid arthritis. Nephrol Dial Transplant.

[28] Maruotti N, Corrado A, Gaudio A, Cantatore FP (2009). Membranous nephropathy in rheumatoid arthritis: a case report. Clin Exp Rheumatol.

[29] Kaushik P, Rahmani M, Ellison W (2011). Membranous glomerulonephritis with the use of etanercept in ankylosing spondylitis. Ann Pharmacother.

[30] Wang YL, Chou FC, Chen SJ, Lin SH, Chang DM, Sytwu HK (2011). Targeting pre-ligand assembly domain of TNFR1 ameliorates autoimmune diseases - an unrevealed role in downregulation of Th17 cells. J Autoimmun.

[31] Fu SH, Lin MH, Yeh LT, Wang YL, Chien MW, Lin SH, Chang DM, Sytwu HK (2015). Targeting tumour necrosis factor receptor 1 assembly reverses Th17-mediated colitis through boosting a Th2 response. Gut.

[32] Huang YS, Hsieh HY, Shih HM, Sytwu HK, Wu CC (2014). Urinary Xist is a potential biomarker for membranous nephropathy. Biochem Biophys Res Commun.

[33] Wu CC, Lu KC, Lin YF, Chen JS, Huang CF, Chen CC, Lin SH, Chu P, Sytwu HK (2012). Pathogenic role of effector cells and immunoglobulins in cationic bovine serum albumin-induced membranous nephropathy. J Clin Immunol.

[34] Wu CC, Lu KC, Lin GJ, Hsieh HY, Chu P, Lin SH, Sytwu HK (2012). Melatonin enhances endogenous heme oxygenase-1 and represses immune responses to ameliorate experimental murine membranous nephropathy. J Pineal Res.

[35] Wu CC, Chen JS, Huang CF, Chen CC, Lu KC, Chu P, Sytwu HK, Lin YF (2011). Approaching biomarkers of membranous nephropathy from a murine model to human disease. J Biomed Biotechnol.

[36] Wu CC, Chen JS, Lin SH, Chen A, Sytwu HK, Lin YF (2008). Experimental model of membranous nephropathy in mice: sequence of histological and biochemical events. Lab Anim.

[37] Wu CC, Chen JS, Chen SJ, Lin SH, Chen A, Chang LC, Sytwu HK, Lin YF (2007). Kinetics of adaptive immunity to cationic bovine serum albumin-induced membranous nephropathy. Kidney Int.

[38] Wu CC, Huang YS, Chen JS, Huang CF, Su SL, Lu KC, Lin YF, Chu P, Lin SH, Sytwu HK (2015). Resveratrol ameliorates renal damage, increases expression of heme oxygenase-1, and has anti-complement, anti-oxidative, and anti-apoptotic effects in a murine model of membranous nephropathy. PloS One.

[39] Debiec H, Lefeu F, Kemper MJ, Niaudet P, Deschenes G, Remuzzi G, Ulinski T, Ronco P (2011). Early-childhood membranous nephropathy due to cationic bovine serum albumin. N Engl J Med.

